# The Mechanical Strength of Si Foams in the Mushy Zone during Solidification of Al–Si Alloys

**DOI:** 10.3390/ma10040337

**Published:** 2017-03-24

**Authors:** Jeon Taik Lim, Ji Won Youn, Seok Yong Seo, Ki Young Kim, Suk Jun Kim

**Affiliations:** School of Energy, Materials and Chemical Engineering, Korea University of Technology and Education (KOREATECH), Cheonan 31253, Korea; jeontak0203@koreatech.ac.kr (J.T.L.); longspear@koreatech.ac.kr (J.W.Y.); lllll_vv@koreatech.ac.kr (S.Y.S.); simha@koreatech.ac.kr (K.Y.K.)

**Keywords:** Si–Al alloy, porous materials, solvent refining, centrifugation, mechanical strength

## Abstract

The mechanical strength of an Al-30% Si alloy in the mushy zone was estimated by using a novel centrifugation apparatus. In the apparatus, the alloy melt was partially solidified, forming a porous structure made of primary Si platelets (Si foam) while cooling. Subsequently, pressure generated by centrifugal force pushed the liquid phase out of the foam. The estimated mechanical strength of the Si foam in the temperature range 850–993 K was very low (62 kPa to 81 kPa). This is about two orders of magnitude lower than the mechanical strength at room temperature as measured by compressive tests. When the centrifugal stress was higher than the mechanical strength of the foam, the foam fractured, and the primary Si crystallites were extracted along with the Al-rich melt. Therefore, to maximize the centrifugal separation efficiency of the Al-30% Si alloy, the centrifugal stress should be in the range of 62–81 kPa.

## 1. Introduction

**Figure 1:**
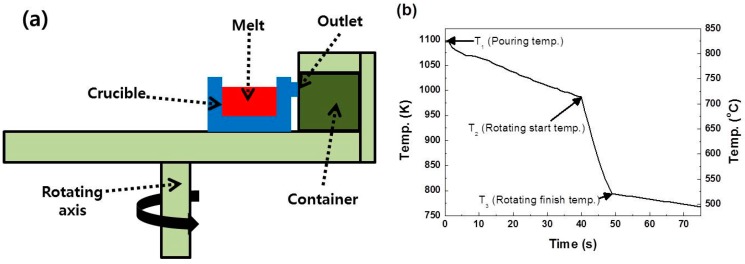
(**a**) Schematic diagram of the novel centrifugal apparatus; (**b**) temperature profile of the melt from the time of being poured into the crucible to the end of the rotation [11].

The solvent refining process has been considered a promising alternative to the Siemens technique or to fluidized bed technique to produce solar-grade Si (SOG-Si) for solar cells [1]. In solvent refining, Si and a solvent element, such as Al, Cu, Fe, or Ni, are melted together and then solidified in a crucible. During the melting process, impurities in Si, such as Mn, Zn, Sn, and Pb, are removed by preferential segregation into the solvent phase. After solidification of the alloy, separating the solvent (metal) phase from the primary Si one is critical in the reduction of impurities in Si. The solvent phase has been separated from the Si by using several techniques, such as conventional centrifugation, electromagnetic separation, and sedimentation [2,3,4,5,6,7,8,9]. The conventional centrifugal separation produces Si with a higher purity in a shorter time than the other methods. In conventional centrifugation, the less dense phase segregates near the rotational axis, and the denser ones are displaced further from the axis [10]. Conventional centrifugation, however, cannot be applied in the separation of phases with nearly identical densities, such as Si and Al. In our previous report, we designed a novel centrifugation apparatus to solve this problem. This apparatus uses the difference in solidification temperatures of each phase (see Figure 1) [11,12,13].

First, primary Si crystallizes while the Si-metal alloy placed in a crucible solidified (from T_1_ to T_2_ in Figure 1b), and then, the centrifugal force pushed the remaining metal-rich melt outside the crucible (from T_2_ to T_3_). After the separation process, a porous Si foam remains in the crucible. Using the novel centrifugal separation technique, we successfully obtained high-purity primary Si from Al–Si–Fe alloys and Al–Si hypereutectic alloys [14,15]. For the novel centrifugation, processing variables should be carefully controlled to maximize the separation efficiency, i.e., the weight ratio between Si and Al in the Si foam. The Al remaining in the foam can be easily removed after additional purification steps, such as solidification refining and acid leaching [16,17,18,19,20,21,22,23,24]. The critical processing variables of this centrifugation procedure are the rotation start temperature (RST) and the rotational speed (RS). The RST, along with the application of the lever rule to the phase diagram, indicates the amount of primary Si that crystallizes. In the present study, the RST was held at 993 K to study the effect of the RS on the separation efficiency. Faster RSs lead to a higher gravitational acceleration, which exerts a greater force on the remaining melt to be forced from the crucible. When the RS is too fast, however, a great pressure may be exerted on the network as well. If the centrifugal stress is higher than the mechanical strength of the Si foam, the Si foam may fracture, and the Si crystallites driven outward along with the Al-rich melt. This results in lower separation efficiency. Thus, the mechanical strength of Si foams should be analyzed to increase the separation efficiency. The purpose of this study is to evaluate the mechanical strength of the Si foams during centrifugation, with a temperature range from 850 K to 993 K, that is, from the eutectic temperature to the RST. The evaluation will help to improve the efficiency in separating Si from an Al-30% Si alloy using the centrifugation apparatus. Various in situ measurement machines, such as nano-indentation machines or other professional test machines, have been adapted to measure the mechanical strength at high temperatures [25,26,27,28]. Instead, here we estimate the mechanical strength of Si foams at high temperatures by analyzing their microstructure and Si concentrations after centrifugation. Centrifugal stress exerted on the foams was calculated based on the geometry of the centrifugation apparatus, and the centrifugal stress was controlled by changing the RS.

## 2. Experimental

A schematic drawing of the novel centrifugal separation apparatus is provided in Figure 1. The apparatus consists of a crucible and a container. The Si foams consist of primary Si crystallites remaining in the crucible, with the Al-rich melt being pushed into the outer container during rotation. For each separation process, 2 kg of an Al-30% Si melt, prepared in a furnace held at 1195 K, and about 50 g of the alloy melt were used. The temperature of the melts in the crucible decreased from 1195 K to 993 K (RST) at a rate of 3 K/s. The RS was in the range of 300 revolutions per minute (rpm) to 800 rpm. All operations from melting the mother alloys to centrifugation were performed for 10 s in air. After separation, Si foam was leached for 2 h at room temperature (RT) to obtain Si flakes. Leaching solution was aqua regia, the mixture of 36% hydrochloric acid and 63% nitric acid (3:1 *v*/*v*). Recovery-1 is the weight ratio of the Si foam to its initial melt, and Recovery-2 is the weight ratio of the Si flakes, obtained after acid leaching the Si foam, to the melt. The morphologies of the Si foam and the Si crystallites were observed by optical microscopy (OM, GX71, Olympus, Seoul, Korea), scanning electron microscopy (SEM, JSM 7500F, JEOL, Seoul, Korea) coupled with energy dispersive X-ray spectroscopy (EDS, JSM 7500F, JEOL, Seoul, Korea), and engineering computed tomography (CT, Rayscan 250, Walischmiller, Markdorf, German). X-ray fluorescence (XRF, EDX-720, Shimadzu, Kyoto, Japan) was used for the elemental analysis of the Si foams. Open pores were determined by measuring the densities and apparent densities of the Si foams. Closed pores were quantified by using the theoretical densities of the Si foams. The theoretical densities of the Si foams were calculated by using both theoretical densities of pure Si (2329 kg/m^3^) and Al (2700 kg/m^3^) and the ratio of Al to Si in a Si foam measured by XRF. Size and aspect ratio of the pores in the Si foams were measured by using ImageJ, image analysis software (version 1.48v) [29]. For compressive tests, the center parts of Si foams were cut to the dimensions of 10 × 10 × 10 mm^3^ using a precision diamond saw. The compressive tests with a static strain rate of 1.25 × 10^−3^/s were conducted at RT using a universal testing machine (H5K-T, Tinius Olsen, Horsham, PA, USA). The compressive strength in this study was defined as the maximum strength just before the breakdown of the specimen.

## 3. Results and Discussion

### Recoveries of Foams

**Figure 2:**
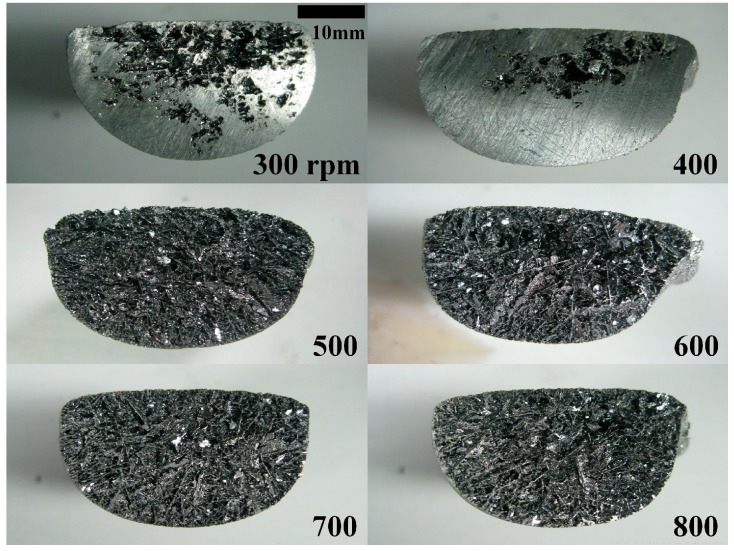
Optical microscopy (OM) images of the cross-sectional areas of the Si foams fabricated by centrifuging an Al–Si alloy at various rotational speeds (RSs) from 300 rpm to 800 rpm.

**Figure 3:**
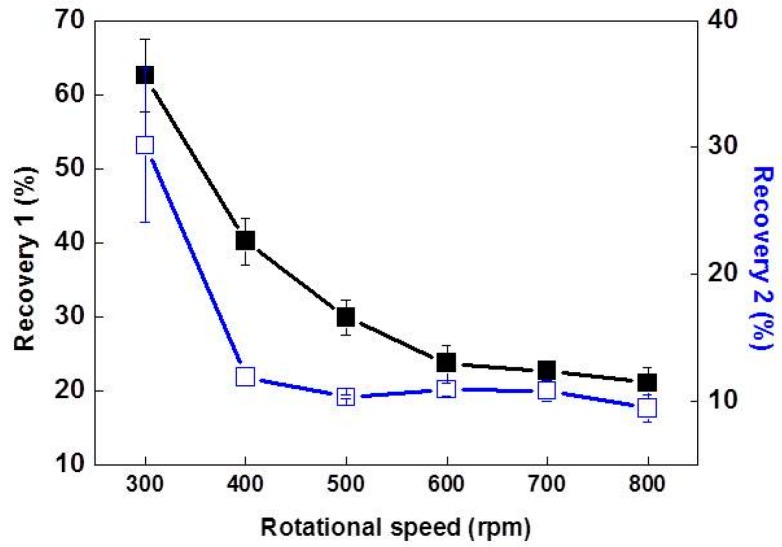
Recovery-1 and Recovery-2 of the foams fabricated at various RSs.

**Figure 4:**
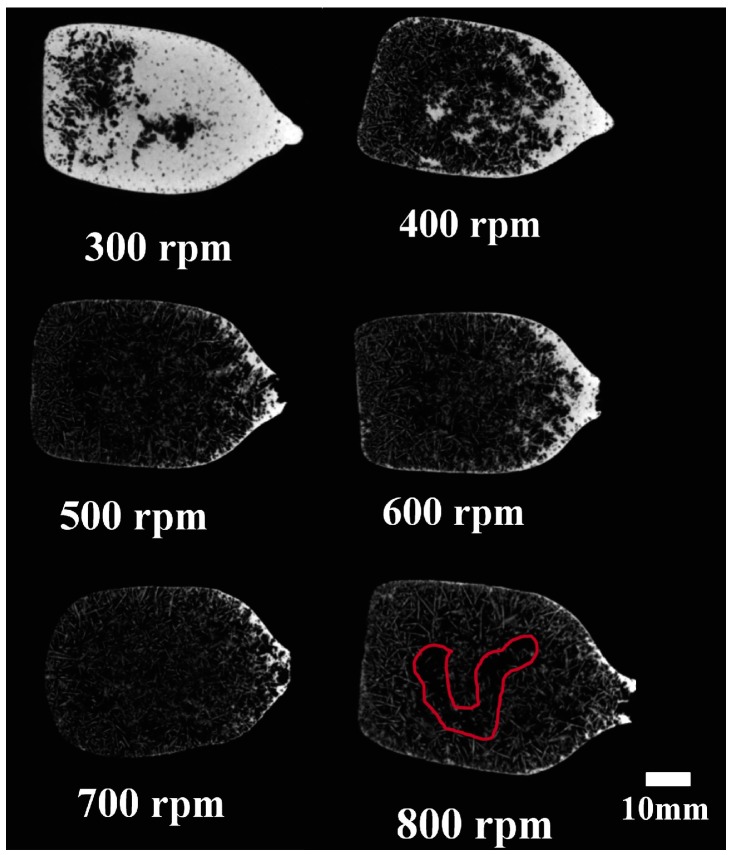
Computed tomography (CT) images of the foams fabricated at various RSs. The area outlined with a red line indicates open structure.

Si foams were produced at various RSs from 300 rpm to 800 rpm. Higher RSs led to the removal of a larger amount of Al-rich melt, based on the analysis of the cross-sectional areas of the foams with OM after cutting them in half, as shown in Figure 2. Measurement of recovery confirmed that the amount of Si in the foam decreased asymptotically to about 10%, which is close to the Si concentrations at the RST. The recoveries were carefully measured, and they are presented in Figure 3. As the RS increased, Recovery-1 reduced and saturated at about 20%. Possibly, the maximum RS of the centrifuge, which is 800 rpm, was not high enough to remove the Al-alloy melt effectively. On the other hand, Recovery-2 was comparable to 10% when the RS was in the range of 500 rpm to 700 rpm. A relatively lower Recovery-2 at 800 rpm with broader standard deviation clearly confirmed that primary Si crystallites were fractured and removed together with the Al-rich melt during centrifugation. Average Recovery-2 of the foam fabricated at 800 rpm (9.4%) is lower than Recovery-2 of the foams fabricated at 500 rpm to 700 rpm (10.3%–10.9%), and about 1% of the primary Si crystallites were additionally removed at 800 rpm. This is because the centrifugal force exerted at 800 rpm was higher than the mechanical strength of the foam. The lower mechanical strength of the foam at high temperatures was also supported by the CT images, as shown in Figure 4. The foam fabricated at 800 rpm exhibited a more open structure at the core in comparison to the foams fabricated at lower rpms. This indicates that primary Si crystallites were removed by centrifugal force, which fractured the Si crystallites from the Si network.

It is of importance to note that the relatively higher Recovery-2 of the foams fabricated at 300 rpm and 400 rpm was due to the larger amount of Al remaining after acid leaching. This was confirmed by an elemental analysis with XRF, as summarized in Table 1. The larger amount of Al remaining in the foams fabricated at 300 rpm and 400 rpm was also confirmed by analysis with CT, as shown in Figure 4. Additionally, open structures were observed near the core of the Si foams when the RS ranged from 500 rpm to 700 rpm. This may be because, during centrifugation, the temperature at the core was higher than that at the outer surface. Due to the higher temperature at the core, the formation of loosely connected Si crystallites resulted. When the centrifugal force was sufficient to force the Al alloy melt from the crucible, the primary Si crystallites near the core were also removed. Because of this, the porosity near the core was similar when the RS ranged from 500 rpm to 700 rpm. In addition, the comparable Recovery-2 of these RSs supported that the Si crystallites were removed along with the Al-alloy melt as it was forced from the crucible. 

**Figure 5:**
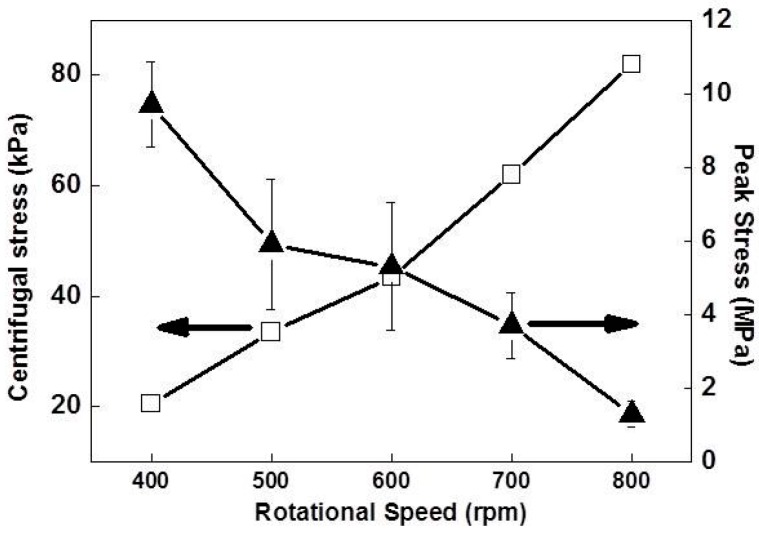
Comparison between the peak stresses measured from compressive tests and the calculated centrifugal stresses. The peak stress is the point that is followed by a sharp decrease of stress in the compressive test.

The estimated mechanical strength of the Si foams during centrifugation in the temperature range of 850–993 K was in the range of 62–81 kPa, which is about two orders of magnitude lower than the mechanical strength of the foam at RT. The mechanical strength of Si foams during centrifugation was estimated by comparing the data sets of the recoveries and the microstructures of Si foams (discussed above) to the centrifugal stresses generated by centrifugal force. As shown in Figure 3, relatively low Recovery-2 of the foam fabricated at 800 rpm compared to the ones fabricated at 600 rpm or 700 rpm was direct evidence of the fracturing of the Si crystallites. The fracturing was attributed to a mechanical strength lower than the centrifugal stress at 800 rpm. Average centrifugal stresses, depending on the rpm, were numerically calculated according to previous reports [30,31] and provided in Figure 5. P exerted by rotation is described by Equation (1):

(1) $$P=\frac{1}{2}\rho_{m}\omega^{2}r^{2}$$

where *P* is the pressure at *r*, i.e., the length from the axis of rotation to the center of the Si foam, $\rho_{m}$ is the density of the melt, and *ω* is the angular velocity. For the calculation, *r* was set at 9.75 cm based on the geometry of the centrifuge used in this study. The centrifugal stress was almost solely exerted on the foam, which stayed in the crucible once the melt remaining in the crucible was removed. When the centrifuge started rotating, the remaining melt was promptly removed to the container within a few seconds. This is because the centrifugal force was much greater than the capillary force at the outlet channel. The capillary force restrained the melt within the crucible before centrifugation. 

**Figure 6:**
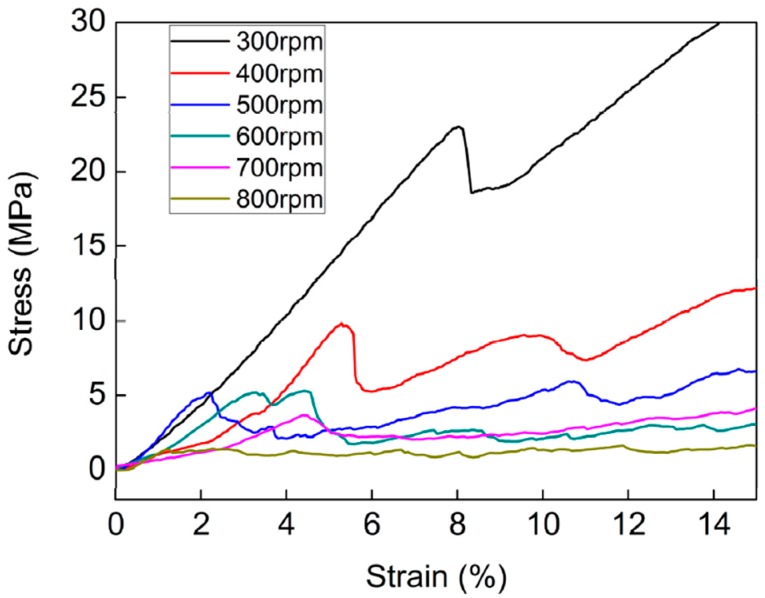
Stress–strain curves of the foams fabricated at various RSs as analyzed by compressive tests.

The mechanical strengths of the Si foams fabricated at various RSs were measured at RT by compressive tests, as shown in Figure 6. They were compared to the centrifugal stresses at various RSs. As the stress increases linearly during the first stage of the test, the Si foam reached a peak value. The peak value indicates the collapsing of the cell edges of the foams, which are connected via an Al-rich phase. After the collapsing of the cell edges, the stress decreases very rapidly immediately afterward. As additional load is applied after the breakdown, cracks propagate in the silicon flakes, and the stress gradually increases due to densification. The peak values, i.e., the mechanical strengths of the foams at RT, were compared to the centrifugal stress exerted by the rpm at which each foam was fabricated in Figure 5. The mechanical strength of the foam fabricated at 800 rpm was about 1.3 MPa at RT, and the centrifugal stress at that rpm was 81 kPa, at which the foam was fractured (discussed above). Thus, the mechanical strength of the foam at RST was higher than 62 kPa (exerted by 700 rpm) and lower than 81 kPa, which is about two orders of magnitude lower than the mechanical strength of the foam at RT. At temperatures above the eutectic point, the strength of the Si foam is expected to decrease in comparison to that at RT, according to a previous report [32]. In the literature, the tensile strength of the Al–11.7% Si alloy at RT is 205 MPa and decreases very rapidly (to almost zero) as the temperature increases to its liquidus line. 

The Si foams fabricated at various RSs in the present study shows two different mechanical behaviors from general porous materials. First, the mechanical strength of the Si foam fabricated at 800 rpm was not governed by factors known to control the mechanical strength of general porous materials. According to previous reports, the mechanical strength of general porous materials is governed by several factors such as distribution, size, and the aspect ratio of pores and porosity [33,34]. However, these factors did not seem to contribute to the lower mechanical strength of the Si foams fabricated at 800 rpm more so than those fabricated at 600 rpm or 700 rpm, as shown in Table 2. No apparent differences in the average size of pores or their standard deviations were observed, and the aspect ratios of the pores were in the error range. Instead, the centrifugal pressure at 800 rpm, high enough to fracture Si foams as discussed above, may have reduced the mechanical strength of the Si foams at RT. Thus, the mechanical strength of the Si foams did not follow the generalized mixture rules proposed in the previous report [34]. 

**Figure 7:**
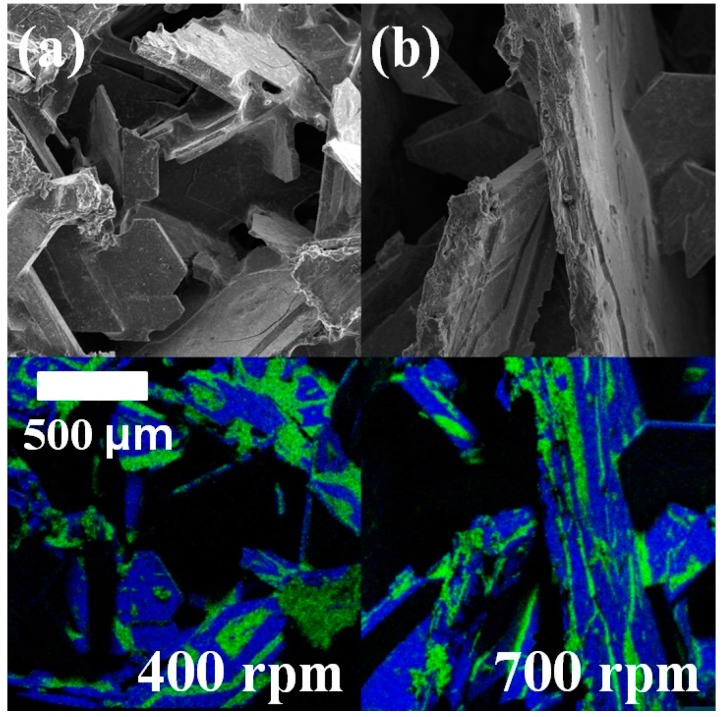
Fracture profiles of the specimens after static compressive tests of the Si foams analyzed with SEM (top) and EDX mapping (bottom, Si-blue and Al-green). The Si foams were fabricated at (**a**) 400 rpm and (**b**) 700 rpm.

One thing should be mentioned here. Most of the average sizes of pores in Table 2 are smaller than their standard deviations. This is due to a few extraordinarily large pores. The wide standard deviation of the pore sizes resulted in the standard deviations of the values of the peak stresses in the range of 0.67 MPa to 3.54 MPa. However, it did not influence the general relationship: the average value of the peak stresses decreased as RPM increased. Second, some of the Si foams do not exhibit a plateau stress during the compressive test. Although, common open-cell foams tend to have a plateau stress [35]; cell edges collapse one by one, while the cells are condensed and accumulated without collapsing during compression. The discrepancy between the stress–strain curves of the foams fabricated in this study and the general ones is because the strength of our foams is strongly dependent on the distribution of the Al-rich phase. The Si foam with a relatively large amount of the Al-rich phase (fabricated at 400 rpm) exhibited that its strain increased as stress increased, as shown in Figure 6. On the other hand, the Si foam with a lower amount of the Al-rich phase (fabricated at 800 rpm) showed a plateau stress during the compressive test. This is because the Al-rich phase produces a ductile fracture, while the silicon phase produces a brittle fracture. As shown in Figure 7, Al (green area in the EDX mapping) exhibited plastically deformed surfaces, while Si (blue area) showed facetted surfaces after fracturing.

## 4. Conclusions

(1) The mechanical strength of Si foams in the mushy zone was estimated to maximize the efficiency of separating Si from the Al–Si alloy using a novel centrifuge.
(2) The estimated mechanical strength of Si foams during centrifugation in the temperature range of 850–993 K was in the range of 62–81 kPa, which is about two orders of magnitude lower than the mechanical strength of the foam at RT (1.3–3.8 MPa).
(3) To maximize the separating efficiency of Si from an Al-30% Si alloy, the optimal RS must exert a centrifugal stress in the range of 62–81 kPa.

## Figures and Tables

**Table 1:** Weight % of Al and Si in the foams fabricated at various RSs, as measured by X-ray fluorescence (XRF).

RS (rpm)	Al (wt %)	Si (wt %)
300	52.4	46.9
400	41.4	58.2
500	37.6	62.1
600	36.4	63.2
700	31.7	67.6
800	26.6	72.6

**Table 2:** Peak stresses and porosities of Si foams fabricated at various RSs and average area and aspect ratio of the pores exist in the Si foams.

Properties		RS (rpm)					
		300	400	500	600	700	800
Peak stress(MPa)		23.3 ± 2.44	9.7 ± 2.32	5.9 ± 3.54	5.3 ± 3.46	3.7 ± 1.77	1.3 ± 0.67
Porosity	Open pore	0.16	0.46	0.64	0.76	0.77	0.7
	Closed pore	0.25	0.10	0.04	0.02	0.01	0.03
Average size of pore (m^2^) × 10^3^		3.16 ± 7.80	6.55 ± 3.23	1.30 ± 2.95	1.94 ± 2.35	1.69 ± 2.40	1.28 ±2.09
Aspect ratio of pores		2.17 ± 1.00	2.2 ± 0.93	2.12 ± 0.92	2.24 ± 0.98	2.36 ± 1.02	2.35 ± 1.21
